# Comparison of an online adaptation of the Autism Diagnostic Observation Schedule-2 with its in-person version in an adult autism diagnostic service

**DOI:** 10.1192/bjo.2023.24

**Published:** 2023-03-06

**Authors:** Charlotte E. Blackmore, Alexandra Nolan, Vladimira Stoencheva, Natalie Greenwood, Natasha Liu-Thwaites, Stefanos Maltezos, Grainne M. McAlonan

**Affiliations:** Department of Forensic and Neurodevelopmental Sciences, Institute of Psychiatry, Psychology & Neuroscience, King's College London, London, UK; and Adult ADHD and Autism Service, Croydon Adult and Behavioural & Developmental Psychiatry Directorate, South London and Maudsley NHS Foundation Trust, London, UK; Adult ADHD and Autism Service, Croydon Adult and Behavioural & Developmental Psychiatry Directorate, South London and Maudsley NHS Foundation Trust, London, UK

**Keywords:** Autism spectrum disorders, autism, Autism Diagnostic Observation Schedule, diagnosis, telehealth

## Abstract

**Background:**

Restrictions on in-person assessments during the COVID-19 pandemic were a challenge for an adult autism diagnostic service receiving over 600 referrals annually. The service sought to adapt the Autism Diagnostic Observation Schedule (ADOS-2) for online administration.

**Aims:**

To investigate whether an online adaptation of the ADOS-2 performed comparably to the in-person ADOS-2. To obtain qualitative feedback from patients and clinicians regarding experiences of the online alternative.

**Method:**

Online ADOS-2 assessments were completed for 163 referred individuals. A matched-comparison group comprised 198 individuals seen for an in-person ADOS-2 assessment prior to COVID-19 restrictions. A two-way analysis of variance (ANOVA) was run to explore any effect of assessment type (online or in-person ADOS-2) and gender on total ADOS score. Qualitative feedback was collected from 46 patients and 8 clinicians involved in diagnostic decision-making after the online ADOS-2 assessment.

**Results:**

A two-way ANOVA found no significant effect of assessment type or gender and no assessment type × gender interaction effect on total ADOS score. Qualitative feedback suggested that only 27% of patients would have preferred an in-person assessment. Nearly all clinicians reported gains from offering an online alternative.

**Conclusions:**

This is the first study to examine an online adaptation of ADOS-2 within an adult autism diagnostic service. It performed comparably to the in-person ADOS-2, making it a viable alternative when in-person assessments are not possible. As this clinic group has high rates of comorbid mental health difficulties, we encourage further work to determine whether online assessment approaches generalise to other services to increase options for patients and efficiencies for service delivery.

Autism spectrum disorder (ASD) is a pervasive neurodevelopmental disorder characterised by impairment in social interactions and communication and restricted, repetitive patterns of behaviour and interests.^1^ It is a common condition with an increasing estimated prevalence rate of at least 1 in 100 in the UK.^2^ A greater proportion of males than females receive a diagnosis, with a ratio of approximately 3:1.^3^ Standardised diagnostic tools such as the Autism Diagnostic Observation Schedule-2 (ADOS-2) and Autism Diagnostic Interview-Revised (ADI-R) are recommended to support the clinical assessment of ASD,^4^ but the diagnosis is ultimately a clinical decision. In adult ASD diagnostic services, the ADI-R is not always possible as there may be no appropriate childhood informant. This has led to an increased reliance on present-state assessments such as the ADOS-2 (module 4). The ADOS-2 is a semi-structured standardised measure of current communication, social interaction, play/imagination and restricted/repetitive behaviours.^5^ Module 4 of the ADOS-2 is recommended for verbally fluent adults and involves in-person administration, close contact/proximity and the sharing of materials. Restrictions associated with the COVID-19 pandemic meant that many services were forced to temporarily suspend administration of the ADOS-2 to protect patients and clinicians. The use of personal protective equipment (PPE) such as masks and social distancing protocols acted as a barrier to social communication and establishing rapport; thus, in-person ADOS-2 assessments were not possible during the pandemic. This placed additional pressure on adult ASD services in the UK, which already had extensive waiting lists. Hence, there was an urgent need for ADOS-2 alternatives and/or adaptations to continue to inform clinical diagnostic assessment.

The effectiveness of using telehealth across different healthcare specialties has already been well established,^6^ with benefits including improved access to services, reduced waiting times and reduced costs for both services and patients. There are also benefits related to risk management (for example, in forensic services). There is preliminary evidence to support the use of telehealth to conduct ASD assessments, including video observations, web and mobile tools, and phone interviews.^7,8^ A study found high classification accuracy, sensitivity and specificity when the ADOS-2 algorithm was applied to informal and unstructured videos of children.^9^ However, using video footage in this way does not allow for a comprehensive assessment of ASD behaviours according to the ADOS-2 protocol^10^ and leads to several ‘not applicable’ algorithm scores.

There is also limited research on the use of the ADOS-2 when administered online. One study evaluated its use through interactive videoconferencing compared with the in-person assessment in children aged 3–5 years.^11^ They found little difference in interrater agreement between ratings on ASD measures scored using videoconferencing and in person, and the clinicians were consistent with one another in all but one case when assigning a diagnosis, regardless of the set up. However, this study used module 1 of the ADOS-2 and was administered by parents who are not trained in the ADOS-2, which affects the standardisation of the assessment. In adult services, there is often no other person in the patient's home, and with COVID-19 restrictions, contact with other households was restricted, so conducting the ADOS-2 using another person guided by a clinician was not an option.

To our knowledge, there has only been one study looking at the utility of administering the ADOS-2 to adults remotely, using an online platform.^12^ Findings indicated that the ADOS-2 administered online had high levels of usability and reliability compared with the conventional in-person ADOS-2. However, this study, using a repeated measures design, was based on a small research cohort of adults with an existing diagnosis of ASD. It is still unclear how an ADOS-2 administered online would be feasible, acceptable and useful when adopted in a large diagnostic service. In addition, given evidence that there are challenges regarding ASD diagnostic processes in females,^13,14^ it is important to consider any potential influence of gender when adapting an instrument for online use. The current study therefore aimed to compare how an online-administered ADOS-2 (module 4) assessment performed relative to previous in-person ADOS-2 administration. Specifically, we used analysis of variance (ANOVA) to test the predictions that there would be:

- no difference in algorithm scores between patients diagnosed with ASD who received an online-delivered ADOS-2 and those who received an in-person ADOS-2
- no difference in algorithm scores between patients not diagnosed with ASD who received an online-delivered ADOS-2 and those who received an in-person ADOS-2
- no influence of gender on algorithm scores acquired using online or in-person administration.

Finally, we examined the qualitative experiences of both patients and clinicians in attending these remote assessments by conducting a brief survey to gain feedback and thematic analysis of their answers.

## Method

### Participants

The sample included 361 patients seen at the Adult ADHD and Autism Service, South London and Maudsley NHS Foundation Trust. This is a specialist national tertiary service that accepts referrals from across the UK for suspected ASD. The sample included 163 patients who had an online ADOS-2 assessment between August 2020 and February 2021 and 198 in the comparison group who had an in-person ADOS-2 assessment between May 2014 and February 2020. Patients who completed an online ADOS-2 were matched by gender and, as closely as possible, by age to a patient who had previously been seen in the service for an in-person ADOS-2. Further matching in terms of educational performance, socioeconomic status (occupation) and location of residence was not attempted; however, the patients assessed by our online adaptation of the ADOS-2 had been referred prior to the pandemic. Thus, it is likely that their background demographic characteristics were not influenced by the pandemic itself as referral routes were the same for those who had an in-person ADOS-2 assessment.

### Measures

ASD diagnosis was made by a clinician using the ICD-10 criteria and information gathered from the ADI-R and/or in-person or online-administered ADOS-2.

### Autism Diagnostic Observation Schedule-2 (ADOS-2)

The ADOS-2 is a semi-structured standardised measure of current communication, social interaction, play/imagination and restricted/repetitive behaviours.^5^ Experienced clinicians administered the in-person ADOS-2 using module 4 (for verbally fluent adults).

### Online ADOS-2

#### The process of development

In line with government guidelines and to ensure the safety of our patients, it was no longer possible to administer in-person assessments during the COVID-19 outbreak. There were no available alternatives to the ADOS-2 that were appropriate for adult cohorts at the time. Clinicians set up a ‘virtual ADOS’ working group made of up of three experienced neurodevelopmental assessors who administer ADOS-2 in the service and representatives from psychiatry, psychology and management. We reached consensus that an online version of the ADOS-2 would be possible, using the online platform Microsoft Teams, as the materials used in module 4 with verbally fluent adults could be transformed into Microsoft Teams compatible versions. Minimal amendments were made to facilitate online administration of the ADOS-2. The ‘Telling a Story from a Book’, ‘Cartoons’ and ‘Description of a Picture’ tasks were transformed into online formats and patients were given guidance on obtaining their own objects for the ‘Creating a Story’ task (full details of amendments are given in the supplementary material S1, available at https://dx.doi.org/10.1192/bjo.2023.24). Patients were sent an information leaflet prior to their online assessment to prepare for the online ADOS-2. This includes information about what device to use, how they should set up their environment before the call and what equipment they need.

#### Online ADOS-2 pilot

Specialist neurodevelopmental assessors tested the viability of the online ADOS-2 by administering the assessment on each other to check its initial usability. It was then trialled on several patients and initial feedback indicated that the outcomes closely matched those of the in-person ADOS-2. Consensus was reached between the three experienced clinicians administering the ADOS-2 that the online ADOS-2 was a useful tool for assessing current ASD symptomology. Coding of all behaviours was possible, apart from ‘unusual eye contact’ (a generic problem with online assessments).

#### Output: coding and report writing

Qualitative reports of observations were provided (as with the in-person ADOS-2) but the scores were omitted. The reports were available for the patient in the appendix of their main diagnostic report. A coding sheet with algorithm scores was provided for the clinicians’ information only. This included a table of all codes and a summary of whether the patient would have scored above or below the threshold for ASD. The only algorithm item that was unable to be coded was ‘B1 Unusual eye contact’ and this was reported as ‘one item could not be scored’ in the algorithm. However, a qualitative description of use of eye contact was provided in the report (for example, based on eye gaze during social interactions in relation to the screen/camera).

#### Qualitative findings

Feedback was collected from patients regarding whether they would have preferred an in-person assessment and their reasoning behind their answer. Clinicians who use the ADOS-2 as part of formulating their diagnosis were also surveyed regarding their apprehensions before and after the online ADOS-2 was rolled out and their views on its utility. Thematic analysis was run on the qualitative responses from the feedback.

### Data analysis

Group differences in demographic data, including age and gender, were analysed with independent-samples *t*-tests and chi-squared tests.

A two-way ANOVA was performed to analyse the effect of assessment type (online or in-person ADOS-2) and gender on total ADOS score. Independent-samples *t*-tests were used to further explore the data where appropriate and to understand within groups how total algorithm and/or subdomain scores differed in those diagnosed or not diagnosed with ASD.

The total ADOS scores can be converted to a Calibrated Severity Score (CSS^15^) which provides an overall symptom severity on a scale from 1 to 10. Therefore, for completeness, we also performed a two-way ANOVA to analyse the effect of assessment type (online or in-person ADOS-2) and sex on total CSS with *post hoc t*-tests where appropriate.

### Ethical standards

The Behavioural Developmental Psychiatry Clinical Academic Group within the South London and Maudsley NHS Foundation Trust reviewed the project and provided approval under the Trust Clinical Audit, Service Evaluation and other Quality Improvement Projects governance procedures regarding the anonymised use of clinical records. In addition, ethical approval for retrospective access to the anonymised clinical records database was provided by the Dulwich subcommittee of the National Research Ethics Committee, Health Research Authority, UK (reference 18.LO.0354). Verbal or written consent was obtained from patients and clinicians for their data to be used for research.

## Results

### Characteristics of participants

The online ADOS-2 group consisted of 163 patients (92 females and 71 males; Table 1). The mean age was 37 years (range 18–66 years). Of this group, 130 (80%; 76 females and 54 males) were diagnosed with ASD and 33 (20%; 16 females and 17 males) did not receive an ASD diagnosis.

**Table 1:** Participant characteristics

	Online ADOS-2	In-person ADOS-2	Test statistic	*P*
Sex, *n* (%)				
Male	71 (44.4)	89 (44.9)	χ² = 0.07	0.8
Female	92 (55.6)	109 (55.1)		
Mean age, years (s.d.)	36.8 (12.5)	36.2 (12.0)	t = −0.4	0.7
ASD diagnosis, *n* (%)				
ASD diagnosed	130 (79.8)	154 (77.8)	χ² = 0.2	0.7
ASD not diagnosed	33 (20.2)	44 (22.2)		

ADOS-2, Autism Diagnostic Observation Schedule-2; ASD, autism spectrum disorder.

The in-person ADOS-2 group consisted of 198 patients (109 females and 89 males). The mean age was 36 years (range 18–66 years). Of this group, 154 (78%; 92 females and 62 males) were diagnosed with ASD and 44 (22%; 17 females and 27 males) did not receive an ASD diagnosis.

### Main effects and interaction of assessment type and gender on total ADOS score

A two-way ANOVA was performed to analyse the effect of assessment type (online or in-person ADOS-2) and gender on total ADOS score. The two-way ANOVA revealed that there was no significant main effect of assessment type (*F*(1, 357) = 0.8, *P* = 0.4) or gender (*F*(1, 357) = 3.2, *P* = 0.1) and no assessment type × gender interaction effect (*F*(1, 357) = 0.6, *P* = 0.4). A two-sample *post hoc t*-test confirmed no evidence of a difference in total online ADOS score and total in-person ADOS score (*t*(359) = 1.0, *P* = 0.3) across the two groups (Table 2). Independent-samples *t*-tests also confirmed no evidence of a difference in subdomain scores for those who received an online ADOS-2 compared with the in-person ADOS-2.

**Table 2:** Online ADOS-2 and in-person ADOS-2 total and subdomain scores

ADOS algorithm domain	Online ADOS-2, mean (s.d.)	In-person ADOS-2, mean (s.d.)	*t*	*P*
Total ADOS score	6.5 (4.1)	7.0 (3.8)	1.0	0.3
Communication score	1.9 (1.8)	1.8 (1.7)	−0.6	0.5
Social interaction score	4.7 (2.6)	5.2 (2.8)	1.7	0.1

ADOS-2, Autism Diagnostic Observation Schedule-2.

### Within-group analyses of scores acquired in patients who did or did not receive an ASD diagnosis

**Fig. 1:**
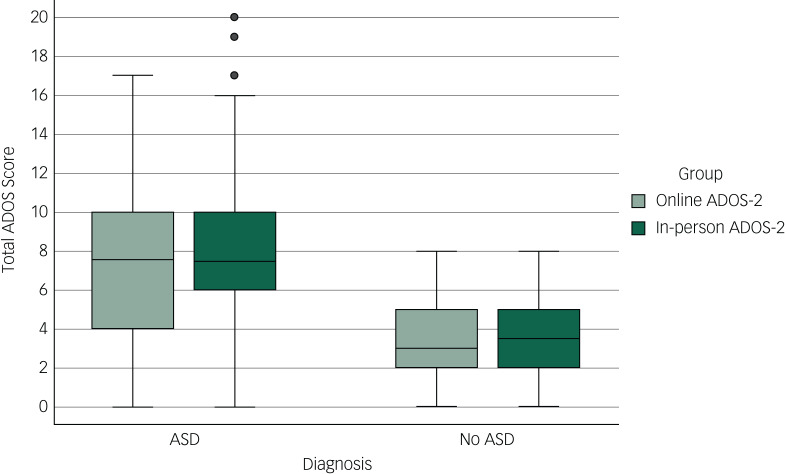
Mean total scores on the Autism Diagnostic Observation Schedule-2 (ADOS-2) for the online ADOS-2 group and in-person ADOS-2 group, split by whether patients did or did not receive a diagnosis of autism spectrum disorder (ASD).

When the samples are split into patients who were diagnosed with ASD and who were not diagnosed, there is no difference in total ADOS score between those who had an online ADOS-2 and those who had an in-person ADOS-2 (Table 3; Fig. 1). There was also no significant difference in the communication domain scores between those who had an online ADOS-2 and an in-person ADOS-2 in both the ASD diagnosed and ASD not diagnosed groups. When looking at those diagnosed with ASD, there was a significant difference in social interaction score between the online and in-person groups, with the in-person ADOS-2 group mean significantly higher. This is probably explained by the item ‘B1 unusual eye contact’ not being scored on the online ADOS-2 adaptation, which removed the option of a score of ‘0’ or ‘2’ from each online observation.

**Table 3:** Online ADOS-2 and in-person ADOS-2 scores for patients who did and did not receive an ASD diagnosis

Group	ADOS algorithm domain	Online ADOS-2, mean (s.d.)	In-person ADOS-2, mean (s.d.)	*t*	*P*
ASD diagnosed	Total ADOS score	7.4 (4.1)	7.9 (3.7)	1.2	0.1
	Communication score	2.2 (1.8)	2.0 (1.7)	−1.6	0.3
	Social interaction score	5.2 (2.6)	5.9 (2.6)	2.2	0.02*
ASD not diagnosed	Total ADOS score	3.3 (1.9)	3.7 (2.2)	0.9	0.2
	Communication score	1.0 (1.3)	0.7 (1.0)	1.4	0.09
	Social interaction score	2.7 (1.4)	2.6 (1.4)	0.1	0.5

ADOS-2, Autism Diagnostic Observation Schedule-2.

* *P* < 0.05.

### Within-group analysis of scores acquired in patients in the online ADOS-2 group and in the in-person ADOS-2 group

In both the online ADOS-2 group and the in-person ADOS-2 group, those who were diagnosed with ASD had a significantly higher total ADOS score than those who were not diagnosed with ASD (supplementary material S2). This was also shown for the communication score and the social interaction score.

### Calibrated severity score

The total ADOS score for each patient was converted to a Calibrated Severity Score (CSS) (Supplementary material S3 and S4). A two-way ANOVA was performed to analyse the effect of assessment type (online or in-person ADOS-2) and gender on CSS. The two-way ANOVA revealed no significant main effect of assessment type (*F*(1, 357) = 0.3, *P* = 0.6). There was a significant main effect of gender (*F*(1, 357) = 5.1, *P* = 0.03). Overall, males had a significantly higher total CSS than the females (3.9 and 3.4 respectively). There was no significant assessment type × gender interaction found (*F*(1, 357) = 0.6, *P* = 0.4), supporting that there was no effect of assessment type on total ADOS score.

### Qualitative feedback

#### Patient feedback

We asked 46 patients for their feedback after their online ADOS-2 assessment. We asked ‘Would you have preferred to have done your assessment in person?’. A majority of 59% (27) reported that they would not have preferred to have had their assessment in person, 28% (13) reported they would have preferred to have had their assessment in person and 13% (6) had no preference.

Patients were also asked for reasons behind their answers. Responses were transcribed, coded and a thematic analysis was carried out. The analysis revealed four main themes: convenience, environment, using technology and human contact (Supplementary material S5).

##### Convenience

Patients commented that the online ADOS- 2 was more time efficient, as it required less travelling and prior organisation (including planning for other people to support them in attending their appointment). One comment suggested that this could improve attendance rates.

##### Environment

Patients generally reported a preference for being in the home environment, as opposed to the hospital (for example, because they felt more relaxed and had familiar things around them). However, there were some comments on having limited space for the tasks, confidentiality concerns (when living in shared accommodation) and increased distractibility.

##### Using technology

Patients suggested that the use of technology could be stressful, including concerns about how reliable it was. One commented on the need to seek technical help from a relative to set up the call. Other comments indicated that mostly patients felt competent in video calls from their experience so far during the pandemic (for example, working from home) and did not see them as a barrier to their assessment. Some patients expressed uncertainties regarding the clinician's ability to pick up on non-verbal behaviours during online assessments and other comments indicated that a larger screen size facilitated a better assessment experience.

##### Social anxiety

Many patients reported that having the option to do their assessment from home and therefore having less in-person contact made the process less stressful and it felt ‘safer’. Some comments indicated that the stress of in-person contact could lead to differences in behaviour, which could affect assessment outcomes.

##### Personal connection

Some patients’ comments indicated that conducting assessments online affected the perceived ‘connection’ between them and the examiner, which for one person was more difficult when talking about particularly emotive topics. One patient commented ‘I don't think it would have been different, but it might have felt different’.

#### Clinician feedback

In total, eight clinicians who use the outcomes (reports and scores) from the ADOS-2 to inform their diagnostic assessments were surveyed. Seven clinicians were asked whether they had initial apprehensions, prior to the online ADOS-2 being rolled out in clinics. Only one clinician (14.3%) said ‘yes’ to being initially apprehensive, with five (71.4%) agreeing they were ‘partly’ apprehensive and one (14.3%) responding ‘no’ they were not apprehensive. The one remaining clinician of the eight joined the service during the time the online ADOS-2 was being used so had not experienced the diagnostic assessments when they were carried out in-person.

The novelty of the online ADOS-2, including the lack of data to validate its use, possible technical limitations and the potential for missing clinically important information, accounted for some of these apprehensions. It was also noted that confidence in the experience and abilities of clinicians administering the online ADOS-2 addressed some of these apprehensions and that it was still considered a useful alternative when in-person assessments were not possible. When asked if they continued to have these apprehensions, five (71.4%) agreed ‘no’ they did not, one (14.3%) agreed ‘partially’ and one (14.3%) agreed ‘yes’ they still had apprehensions.

Those who did not continue to have apprehensions commented on the quality of the information obtained in the online ADOS-2, the consistency between online ADOS-2 outcomes and their own observations and confidence in the experience and abilities of clinicians administering the online ADOS-2, including technical proficiencies (such as using adaptations allowing for a wide camera view). Continued apprehensions concerned the impact of the missing code (for eye contact) on outcomes. However, clinicians who did not have apprehensions commented that eye contact was only one part of an assessment and missing this code would not influence them in making a diagnosis.

Finally, clinicians were asked whether they felt that there were any gains from offering the online ADOS-2 as an alternative to the in-person ADOS-2. Of the eight clinicians surveyed, seven (87.5%) responded ‘yes’ and only one (12.5%) responded ‘no’. The gains reported included reducing patient anxiety about planning a journey to the hospital, travelling on public transport and difficulties in waiting areas, so patients would be more at ease in a familiar environment at home. Being flexible in the approach can lead to smarter use of resources, facilities and clinician time, and reach individuals who would not be able to come in for an in-person assessment. The one clinician who reported ‘no’ did not provide elaboration.

## Discussion

This study aimed to investigate whether the performance of an ADOS-2 adapted for online delivery was comparable to the conventional in-person ADOS-2 when generating algorithm scores to support the diagnostic process in an ASD diagnostic service. We also explored the potential influence of patients’ gender on the ADOS-2 outcome generated online or in-person.

For patients diagnosed with ASD after assessment, the only difference in algorithm scores between those who received the online ADOS-2 and those who received the in-person ADOS-2 was in the social communication domain, because ‘unusual eye contact’ could not be scored. For patients not diagnosed with ASD after assessment, we found no difference in algorithm scores acquired using the online ADOS-2 and the in-person ADOS-2. We found no influence of gender on algorithm scores acquired using the online or in-person ADOS-2.

For both the online and in-person ADOS-2 groups, those who received an ASD diagnosis had higher total ADOS score, communication score and social interaction score than those who were not diagnosed with ASD. This suggests that the ADOS-2 module 4 performs similarly in our adult clinic setting, regardless of delivery method.

Owing to the nature of video assessments, it was not possible to score eye gaze on the online ADOS-2 (usually scored 0 or 2). Inspection of the interquartile range indicates that it was wider for those with an ASD diagnosis in the online ADOS-2 group. The first-quartile values were most likely lower in this group because eye contact was not scored. However, there was no effect on assessment type (online or in-person ADOS-2) on overall ADOS total score.

We have previously reported that the patient's gender does not influence ADOS-2 outcomes, when administered in person.^16^ Nevertheless, given other evidence that females face challenges when seeking an ASD diagnosis,^13,14^ we explored whether males scored differently in the online and in-person delivered ADOS-2 and whether females scored differently in the online and in-person delivered ADOS-2. Thus, our study was not designed to address the effect of gender on the ADOS-2 itself.

We found that ADOS-2 scores were no different in males who received an ASD diagnosis following the online or in-person ADOS-2 or in females who received an ASD diagnosis following the online or in-person ADOS-2. Our Calibrated Severity Scores (CSS) did confirm a main effect of gender, with males having higher total CSS than females, as expected from studies using the in-person ADOS-2.^17^ However, there was no assessment type (online or in-person ADOS-2) × gender interaction. Therefore, our online-delivered ADOS-2 was comparable to the in-person ADOS-2 (performing equally well or poorly at assessing females or males). This suggests that neither males nor females were disadvantaged by having an ADOS-2 assessment online rather than in person. We emphasise that our results only speak to ADOS-2 outcomes and that the final decision regarding ASD diagnosis is a clinical one. It was beyond the scope of this study to examine what influences ASD clinical diagnostic decision-making.

When looking at CSS,^15^ our results held. There was no difference in CSS between those who received an online ADOS-2 and those who received an in-person ADOS-2. This was also the case when the groups were split by whether they received an ASD diagnosis or not. There was an effect of gender on CSS: males had a higher CSS than females. There was, however, no assessment type × gender interaction on CSS.

Thus, our work indicates that adaptation to deliver the ADOS-2 online allowed patients to access this component of their assessment despite COVID-19 restrictions on in-person assessments. Having an online assessment can be more convenient for people who struggle with planning and travel, and can reduce stress and anxiety associated with coming to a new setting, seeing new people and the waiting area. As the quantitative data showed no effect of assessment type (online or in-person ADOS-2), the online adaptation of the ADOS-2 could offer patients more choice and reduce some anxiety regarding their assessment.

### Qualitative feedback

This study also gathered some qualitative feedback from both patients and clinicians on this novel approach to the ADOS-2. Many patients (59%) would not have preferred to have had their assessment in person and 13% indicated no preference. The number of clinicians with apprehensions prior to the online ADOS-2 being introduced decreased once it was being used in the clinics.

The feedback provided suggests that introducing the online ADOS-2 has several clear benefits for patients, clinicians and the service as a whole. From the feedback obtained, it seems that having access to the online ADOS-2 may not only improve patient experience, but could improve attendance rates, reduce overall costs and lead to a more accurate observation of impairments associated with ASD (i.e. without elevated anxiety levels inflating scores). The feedback was also useful in terms of shaping the future development of the online ADOS-2, including the conditions required to facilitate a smooth assessment process (for example, camera/screen positioning and set up; access to technology; the use of a larger screen; access to private space). This study specifically addressed an adaptation of ADOS-2 for online delivery and we found that online delivery of this component of our diagnostic pathway for ASD (the ADOS-2 assessment) is comparable to the in-person component. The results should not be taken to suggest that a full online diagnostic pathway is also valid – we did not examine this question. That said, our results do offer additional options (e.g. in hybrid pathways) for patients.

### Strengths and limitations

Our study has several strengths. It advances the field as one of the first studies to explore the use of the ADOS-2 when administered online in a large adult cohort awaiting a diagnostic assessment. Previous studies have used different age groups and therefore different ADOS-2 modules or have used participants who had already been diagnosed with ASD. This study had a large sample of patients awaiting ASD assessment and diagnosis from a real-world out-patient service, and therefore it had direct and immediate patient benefit.

Assessor experience is a strength when considering the validity of our results. The study used assessors who were trained and clinically reliable in administering the ADOS-2, with several years of experience, rather than untrained individuals under a clinician's guidance, as was the case in previous studies.^11^ The development of the ADOS-2 adapted for online delivery was monitored in a working group with various members of the multidisciplinary team and efforts were made to ensure the protocol remained as closely matched to the original as possible. This contrasts with prior studies exploring online methods of assessment. For example, the ADOS-2 algorithm has been applied to pre-recorded videos, but this strategy precludes the use of structured ‘presses’ available in real time and resulted in a greater number of items that could not be coded.^9,10^

Regarding limitations, we adapted module 4 of the ADOS-2 for online delivery as this is most often used in our adult diagnostic service; however, the current findings may not necessarily generalise to other modules. The lower modules of the ADOS-2 (more often used with children) require the use of various objects and materials which would be much harder to set up for online delivery. In these cases, it has been recommended that other diagnostic measures are considered, for example the Brief Observation of Symptoms of Autism (BOSA). The BOSA is often caregiver administered and as our cohort included only adults, it would not have catered for the participants who lived alone, especially as social distancing was in place.

From the feedback obtained, it is possible that the online ADOS-2 would be less appropriate for certain patients, including those with comorbid conditions, those who might struggle with increased distractibility when in the home environment or those who do not have access to, or feel confident using, technology.

Qualitative findings also suggested that the novelty of the online assessment was cause for some apprehension and uncertainty among both patients and clinicians. Thus, it is essential that further research establishing the efficacy of online assessments is carried out to find ways to improve overall confidence in the approach. However, most clinicians agreed that their apprehensions about the online adaptation were alleviated after the experience. The quantitative analyses reported should provide reassurance that the online ADOS-2 performs in a similar way to the in-person ADOS-2 in our clinic process.

### Implications

People with ASD have high rates of comorbid mental health difficulties, and our positive results with the online ADOS-2 might generalise to the use of online assessments in other services, thus having an impact beyond the pandemic to provide a patient-choice driven approach to service delivery.

## Data Availability

The data that support the findings of this study are available from the corresponding author on reasonable request.

## Supplementary material

For supplementary material accompanying this paper visit http://doi.org/10.1192/bjo.2023.24. click here to view supplementary material
