# Supplementary material for: Comparison of an online adaptation of the Autism Diagnostic Observation Schedule-2 with its in-person version in an adult autism diagnostic service
Source: BJPsych Open. 2023 Mar 6;9(2):e51. doi: 10.1192/bjo.2023.24 (PMC10044163; doi:10.1192/bjo.2023.24)
Supplement: Supplementary file 1 [file S2056472423000248sup001.docx]

**Supplementary s1.** Amendments to the ADOS-2 to facilitate online administration

**Task Number:**

1. **Construction Task:** this optional element has been omitted.
2. **Telling a Story from a Book:** we purchased an e-book version of the story used and copied this into a Power Point. We were then able to screen share, taking turns to read the story while still having a good view of the service user.
3. **Description of a Picture:** we made a high-quality scan of the picture and copied into a Power Point. We were then able to screen share and interact with the service user while still having a good view of them.
4. **Conversation and Reporting:** this element remains the same.
5. **Current Work or School:** this element remains the same.
6. **Social Difficulties and Annoyance:** this element remains the same.
7. **Emotions:** this element remains the same.
8. **Demonstration Task:** this element remains the same.
9. **Cartoons:** we made a high-quality scan of the picture and copied into a Power Point. We were then able to screen share and interact with the service user while still having a good view of them.
10. **Break:** this element has been omitted.
11. **Daily Living:** this element remains the same.
12. **Friends, Relationships and Marriage:** this element remains the same.
13. **Loneliness:** this element remains the same.
14. **Plans and Hopes:** this element remains the same.
15. **Creating a Story:** we ask service users to source their own objects for this task from around the home and provide a list of examples. We have our own bag of objects to demonstrate the task in the same way we usually would. There is also the option for the service user to ‘use’ the examiner’s objects (visible on their screen)

**Supplementary s2.** ADOS scores in patients who received and did not receive an ASD diagnosis within the Online ADOS-2 group and In-person ADOS-2 group

| Group | ADOS Algorithm Domain | ASD diagnosed  m (SD) | ASD not diagnosed  m (SD) | t | *p* |
| --- | --- | --- | --- | --- | --- |
| Online ADOS-2 | Total ADOS score | 7.4 (4.1) | 3.3 (1.9) | -8.4 | <0.0** |
|  | Communication score | 2.2 (1.8) | 0.7 (1.0) | -6.8 | <0.0** |
|  | Social Interaction score | 5.2 (2.6) | 2.6 (1.4) | -7.5 | <0.0** |
| In-person ADOS-2 | Total ADOS score | 7.9 (3.8) | 3.7 (2.2) | -9.5 | <0.0** |
|  | Communication score | 2.0 (1.7) | 1.0 (1.3) | -3.6 | <0.0** |
|  | Social Interaction score | 5.9 (2.6) | 2.7 (1.7) | -9.5 | <0.0** |

m: Mean; SD: Standard deviation; **: p<0.01

**Supplementary s3.** Online ADOS and In-person ADOS Calibrated Severity Scores (CSS)

|  | Online ADOS-2  m (SD) | In-person ADOS-2  m (SD) | t | *p* |
| --- | --- | --- | --- | --- |
| Total Group | 3.5 (2.2) | 3.7 (2.1) | 0.7 | 0.5 |
| ASD Diagnosed | 4.2 (2.1) | 4.0 (2.3) | 0.8 | 0.4 |
| ASD Not Diagnosed | 2.0 (0.9) | 1.8 (0.7) | 0.7 | 0.5 |

m: Mean; SD: Standard deviation

**Supplementary s4.** Calibrated Severity Scores (CSS) for the online and in-person ADOS-2 groups, split by those diagnosed with ASD and those who were not diagnosed.
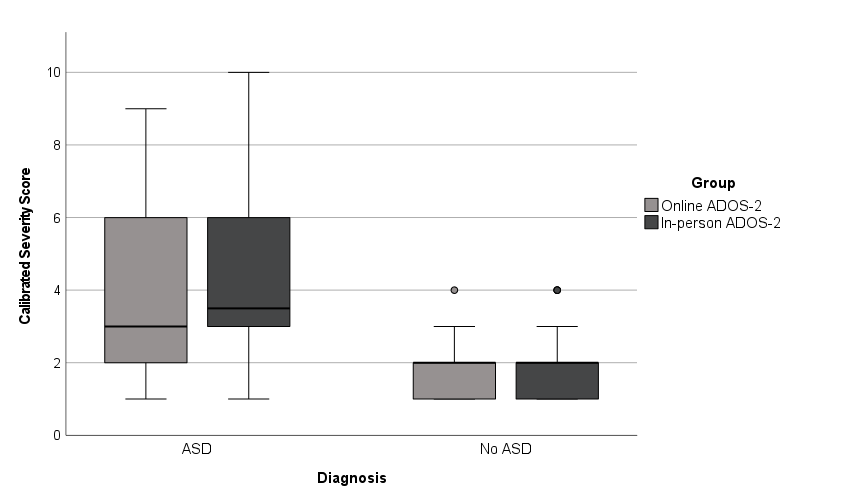


**Supplementary s5: Thematic Analysis from Patient Feedback**

| **Theme** | **Sub-theme** | **Examples** |
| --- | --- | --- |
| Convenience | Time efficient | *“I like that it was virtual because I'm working, and I can go straight back to work after it”* |
|  | Less prior organisation | *“It's a lot easier for me doing it virtually as usually I would need someone to take me to appointments”* |
|  | Less travel considerations | *“I don’t have to go anywhere new, or have to worry about going somewhere… it would have taken a lot longer”* |
|  | Improved attendance rate | *“It’s easier to get out of face-to-face [assessments], this way I had no excuse”* |
| Environment | Preference for being at home | *"I can walk and have familiar things with me [at home]”*  *“I didn't have the nerves I usually do; walking through the hospital and sitting in the waiting room”*  *“I'm more relaxed at home”* |
|  | Space | *“I’d imagine in a clinic room there would be more space [for the tasks]”*  *“Trying to show my objects in front of the screen and sitting in the right position was difficult”* |
|  | Confidentiality concerns | *“I was worried that my flatmates may hear”* |
|  | Increased distractibility | *“My mind is wandering right now because I’m at home”* |
| Using technology | Increased stress | *“The technology is stressful”*  *“Practically, having to set it up […] I was worried it wouldn't work”* |
|  | Technical competency | *“My son helped me set up the laptop […]”*  *It worked very well for me; we are well trained in these [video calls] now. For people who do not have the equipment or are not as technically literate, it might be a problem”*  *“I’ve got used to zoom; it’s made me more open to [online appointments]”* |
|  | Size of screen | *It might have been better having the physical tasks because it was really small [on a phone]”*  *“It was a bit harder because I was using a phone with a small screen”*  *“The book task worked quite well virtually using a large screen”* |
|  | Quality of assessment | *“It feels more removed because I'm on a screen. It's probably easier for you to understand body language [face to face]; I don't know if it's that easy to pick up on a screen”*  *“Analysing behaviour when you see the person is easier”* |
| Social anxiety | Less in person contact | *“I don't have to […] sit in a room with someone face to face, that's stress for me”*  *“It's easier to speak to someone in my own home; I don't really like contact with people”*  *“I guess [online] is better in a certain way because you don't have to deal with people”* |
|  | Feeling safer | *“Meeting someone virtually is a lot easier than meeting someone in person. […] in some ways you feel a bit more confident doing things virtually, there’s a distance and somehow it is safer”.* |
|  | Differences in behaviour | *“I would have problems with face to face […] knowing how to behave, sit and dress”.*  *“I'm quite expressive and wouldn't feel as comfortable [demonstrating exaggerated gesture use] in an office”* |
| Personal connection | Emotive content | *“When asking about my past and things that make me upset it's easier to have that personal connection [when face to face]”* |
|  | Differences in interaction | *“It’s just easier to interact with someone face to face. I don't think it would have been different, but it might have felt different”* |
